# Diet and deprivation in pregnancy: a rat model to investigate the effects of the maternal diet on the growth of the dam and its offspring

**DOI:** 10.1017/S0007114523002210

**Published:** 2024-02-28

**Authors:** Halil Dasgin, Susan M. Hay, William D. Rees

**Affiliations:** The Rowett Institute of Nutrition and Health, The University of Aberdeen, Foresterhill, Aberdeen, AB25 2ZD, UK

**Keywords:** Developmental origins of disease, Adiposity, Metabolism, Hepatic gene expression

## Abstract

The offspring of women in the poorest socio-economic groups in Western societies have an increased risk of developing non-communicable disease in adult life. Deprivation is closely related to the consumption of a diet with an excess of energy (sugar and fat), salt and a shortage of key vitamins. To test the hypothesis that this diet adversely affects the development and long-term health of the offspring, we have formulated two rodent diets, one with a nutrient profile corresponding to the diet of pregnant women in the poorest socio-economic group (DEP) and a second that incorporated current UK recommendations for the diet in pregnancy (REC). Female rats were fed the experimental diets for the duration of gestation and lactation and the offspring compared with those from a reference group fed the AIN-93G diet. The growth trajectory of DEP and REC offspring was reduced compared with the AIN-93G. The REC offspring diet had a transient increase in adipose reserves at weaning, but by 30 weeks of age the body composition of all three groups was similar. The maternal diet had no effect on the homoeostatic model assessment index or the insulin tolerance of the offspring. Changes in hepatic gene expression in the adult REC offspring were consistent with an increased hepatic utilisation of fatty acids and a reduction in *de novo* lipogenesis. These results show that despite changes in growth and adiposity maternal metabolic adaptation minimises the adverse consequences of the imbalanced maternal diet on the metabolism of the offspring.

Inadequate nutrition in early life has been linked to an increased risk of the offspring developing metabolic, cardiovascular and other non-communicable diseases in adult life^(1)^. Data from historical events (e.g. Dutch Hunger Winter, The Leningrad Siege)^(2,3)^ or birth cohorts dating from the 1920s and 1930s (e.g. Helsinki Birth Cohorts)^(4,5)^ suggest that inadequate maternal nutrition due to starvation or poverty was a common theme associated with poor long-term health^(6,7)^. However, over the last 75 years there have been big changes in human diets, especially in developed Western nations, where the prevalence of famine has diminished as changes in agricultural practices and industrial development have increased food production. As a result, the diet of those in the poorest socio-economic groups now contains low-cost, energy-dense, processed food, which is poor in micronutrients^(8,9)^. These diets provide excessive amounts of energy, in the form of fat and refined sugar, but at the same time still suffer from multiple mild-micronutrient deficiencies^(10,11)^. This imbalanced diet creates the phenomenon of hidden malnutrition^(12)^ and may be an important factor in the relationship between social deprivation and an increased risk of ill health in adult life.

Animal studies have proven valuable in investigating the mechanisms underlying the phenomenon known as the developmental origins of health and disease. Restricted fetal and neonatal growth caused by deficiencies of individual key nutrients in the maternal diet (reviewed by^(13–15)^) leads to a range of adverse outcomes including changes in the offspring’s insulin action^(16,17)^, appetite^(18)^ and adiposity^(19,20)^. Similar outcomes have also been reported when animals are fed diets containing excess nutrients, including high-fat diets^(21,22)^ or high-fat diet with additional salt^(23,24)^, sucrose^(25,26)^ or sweetened condensed milk^(27,28)^. However, these approaches fail to emulate the real-life situation and have been criticised for creating extreme and unrealistic imbalances of individual nutrients^(29)^. Additionally, these models do not address the complex interactions between macro- and micro-nutrients. For example, the daily requirement of thiamine is related to energy metabolism, especially the utilisation of carbohydrate, and as a result the requirement changes depending on the carbohydrate content of the diet^(30,31)^. Although severe micronutrient deficiency is rare in Western societies, mild deficiency is common^(32)^ and it is possible that the oversupply of macronutrients, coupled with multiple mild micronutrient deficiencies, may be as serious as a major deficiency or excess of a single nutrient.

The aim of this study was to develop a rodent diet, which reflected the imbalanced diet eaten by pregnant women in deprived Western populations and to use this in an animal model to investigate the long-term consequences for the offspring. The semi-synthetic diet used in these experiments was formulated using information on the diet of pregnant women from the most socio-economically deprived group in Scotland (as defined by the Scottish Index of Multiple Deprivation) described in the study of Haggarty *et al*.^(9)^ By using a semi-synthetic diet, we aimed to overcome the difficulties posed by the inherrent variability of diets based on natural products. A semi-synthetic diet also takes into account the differences in metabolic rate between humans and rodents by adjusting the proportions of micronutrients using the principles of energy density^(33,34)^. As a comparison, we also formulated a second semi-synthetic rat diet, which broadly followed the current UK recommendations for diet in pregnancy, that is, with lower levels of saturated fat, free sugars and salt, increased quantities of PUFA and the recommended micronutrient profile. There are a number of key differences between these two diets and the AIN-93G diet, widely used in experiments with rats and mice, so a third group of animals fed this diet was included as a reference population. This study reports the growth and metabolism of the offspring of dams fed the deprived (DEP), recommended (REC) and AIN-93G (AIN) diets for the duration of gestation and lactation.

## Methods

### Diet formulation

The macronutrient and micronutrient composition of the experimental diets is shown in Tables 1 and 2.

**Table 1.** Diet formula

Component	AIN-93G			DEF			REC		
	g/kg	kJ/kg	% energy	g/kg	kJ/kg	% energy	g/kg	kJ/kg	% energy
Maize starch	398	6646·6	39·5	256·7	4286·9	22·7	469	7832·3	43·4
Maltodextrin	132	2204·4	13·1	70·0	1169·0	6·2	70	1169·0	6·5
Sucrose	100	1670·0	9·9	243·7	4069·5	21·6	39·3	656·3	3·6
Cellulose	50			33·8			60		
Casein	200	3340·0	19·9	98·3	1642·0	8·7	98·3	1642·0	9·1
Gluten				65·6	1094·7	5·8	65·6	1094·7	6·1
l-cystine	3	50·1	0·3	1·5	24·6	0·1	1·5	24·6	0·1
Soyabean oil	70	2639·0	15·7	26·0	980·2	5·2	26	980·2	5·4
Anhydrous milk fat				38·0	1432·6	7·6	19	716·3	4·0
Olive oil				5·0	188·5	1·0	21	791·7	4·4
Lard				40·0	1508·0	8·0	20	754·0	4·2
Beef tallow				51·0	1922·7	10·2	25	942·5	5·2
Maize oil				7·0	263·9	1·4	30	1131·0	6·3
Cholesterol				0·6	21·1	0·1	0·3	11·3	0·1
Choline chloride	2·0			0·6			2·0		
Vitamin mix	10·0*	165·5	1·0	10·0	165·5	0·9	10·0	165·5	0·9
Mineral mix	35·0*	103·5	0·6	35·0	103·5	0·5	35·0	146·0	0·8
Sodium chloride				17·3			7·7		
Total	1000	16 819	100·0	1000·0	18 873	100	999·8	18 057	100·0

* The AIN-93G diet used vitamin and mineral pre-mixes described elsewhere^(72)^.

**Table 2.** Vitamin and mineral pre-mix formula

Vitamin mix (use 10 g/kg)	DEP	REC
	g/kg	g/kg
Nicotinic acid	3·879	3·879
Ca pantothenate	0·989	0·989
Pyridoxine-HCl	0·473	0·473
Thiamin-HCl	0·337	0·337
Riboflavin	0·332	0·332
Folic acid	0·050	0·050
Biotin	0·006	0·006
Vitamin B_12_ (cyanocobalamin) (0·1 % in mannitol)	0·900	0·900
Vitamin E (all-rac-a-tocopheryl acetate)	1·7029	1·702
Vitamin A (all-trans-retinyl palmitate)	0·065	0·065
Vitamin D_3_ (cholecalciferol)	0·0005	0·0005
Vitamin K_1_ (phylloquinone)	0·075	0·075
Powdered sucrose	991·9	991·9
Mineral mix (use 35 g/kg)	DEP	REC
	g/kg	g/kg
Calcium carbonate anhydrous	146·6	164·1
Potassium phosphate monobasic	267·0	116·6
Potassium citrate, tripotassium monohydrate	327·4	388·1
Potassium sulphate	46·6	46·6
Magnesium oxide	29·1	27·9
Ferric citrate	2·924	3·411
Zinc carbonate	1·028	0·705
Sodium meta-silicate·9H_2_O	1·450	1·450
Manganous carbonate	0·0599	0·6300
Cupric carbonate	0·1382	0·1532
Chromium potassium sulfate·12H_2_O	0·2750	0·2750
Boric acid	0·0815	0·0815
Sodium fluoride	0·0635	0·0635
Nickel carbonate	0·0318	0·0318
Lithium chloride	0·0174	0·0174
Sodium selenate anhydrous	0·0087	0·0094
Potassium iodate	0·0471	0·0100
Ammonium paramolybdate·4H_2_O	0·0079	0·0079
Ammonium vanadate	0·0066	0·0066
Powdered sucrose	177·1	249·9

The macronutrient composition of the deprived rodent diet (DEP) was the same as that of women in the most deprived group (decile 10) of the Scottish Index of Multiple Deprivation, containing by weight 16·4 % protein, 17·8 % fat and 59·8 % carbohydrate^(9)^. Protein was provided as a mixture of casein and wheat gluten in the ratio 3:2, to reflect the proportion of animal-based protein (60·3 % of total protein intake) in typical human diets^(35)^. Since casein is poor in sulphur amino acids, an additional 15 mg L-cystine was added per g casein in the diet. Carbohydrates were partitioned between sucrose (free sugar) and a mixture of maize starch and maltodextrin, the latter added to improve the pelleting qualities of the diet. A variety of different fat sources (soyabean oil, anhydrous milk fat, olive oil, lard, beef tallow, maize oil) were used in the diet to mimic the fatty acid profiles of the human diet. The proportions of each component were determined empirically using a Microsoft Excel spreadsheet, changing the quantities of each component until a total of 17·8 g of fat per 100 g of diet comprising 44·3 % SFA, 37·9 % MFA and 17·7 % PUFA was achieved.

The micronutrient content of the diet was based where possible on the data of Haggarty *et al*.^(9)^ The quantities of each micronutrient were adjusted by following principles of nutrient density^(33,34)^. As there was no information on the choline intakes of Scottish women, the choline content was chosen to reflect the lower value for the intake of American women, which was 260 mg/d^(36)^. The final micronutrient composition of the diet is shown in Table 2.

A second experimental diet was formulated, incorporating the current advice of the UK Scientific Advisory Committee on Nutrition^(37)^, namely that 50 % of the metabolisable energy should be derived from carbohydrates (no more than 5 % from free sugars), no more than 35 % of the daily energy should be from fats, with the remainder derived from protein. The total energy intake recommended for women aged 19–34 years is 9·1 MJ/d, increased by 0·8 MJ/d in the last trimester of pregnancy and a further 0·14 MJ/d during lactation. Practical considerations precluded the preparation of more than one experimental diet, and a single diet was formulated providing the equivalent of 9·94 MJ/d, that is, slightly more energy than recommended in the pre-mating and early gestation periods and slightly less during lactation. The fatty acid profile was adjusted to meet the recommendations^(38)^ for fatty acids, namely that SFA, MUFA and PUFA should provide 11, 13 and 6·5 % of daily energy intakes and that in addition, linoleic acid and *α*-linolenic acid should provide at least 1 and 0·2 % of total energy, respectively. The micronutrient composition of the REC diet was based on the Reference Nutrient Intakes^(38)^ for pregnant and lactating women aged between 19 and 50 years and adjusted for energy density. The choline content of the REC diet corresponded to the recommended adequate intake of 450 mg/d for pregnant women^(39)^. Where there were no recommendations, values for the AIN-93G diet were used. The final composition of the REC diet is shown in Tables 1 and 2.

### Animals

All experimental procedures were approved by the ethical review committee of the University of Aberdeen and conducted in accordance with the UK Animals (Scientific Procedures) Act, 1986. Animals were always provided with tap water and housed with an illumination photoperiod of 12L:12D in plastic cages on sawdust bedding under constant conditions of temperature and humidity.

The study was conducted with three separate batches of experimental animals with a total of sixty-six Female Hooded Lister Rats (Charles River UK Ltd Margate, Kent CT9 4LT). On arrival at approximately 10 weeks of age, animals were fed stock diets for an acclimatisation period of approximately 3 d. At the start of the experiment, animals were randomly assigned to one of the three experimental diets, which was fed *ad libitum* for 3 weeks to adapt them to the diet. At 16 d, during the adaptation period, the body composition of the dams was measured by MRI (EchoMRI) as described previously^(40)^. The animals (body weights of 200–250 g) were then mated with normal males of the same strain. After mating animals continued to be fed the experimental diets throughout gestation. The subsequent allocation of the animals is shown in Table 3.

**Table 3.** Allocation of experimental animals

	AIN-93G	DEP	REC
Number of animals			
Animals mated	22	22	22
Pregnant	19	18	18
Killed at D21 of gestation	9	7	8
Failed to nurse litter	3	2	2
Successfully weaned pups	7	9	8
Offspring week 4	19M 20F	20M 23F	22M 25F
Offspring week 10	19M 20F	20M 23F	22M 25F
Offspring week 21	16M 14F	18M 14F	20M 15F
Offspring week 36	9M 10F	10M 7F	13M 12F

On gestation day 21, some animals were anaesthetised with isoflurane and killed by exsanguination. A sample of maternal blood was collected by cardiac puncture. Fetuses were weighed, killed by decapitation and a pooled sample of trunk blood was collected. After discarding the smallest and largest fetuses, four male and four female fetuses were chosen at random from each litter for further dissection. Tissues were frozen in liquid N_2_ and stored at −70°C prior to further analysis.

The remaining animals were allowed to give birth and within 48 h the litters were culled to eight pups, retaining, where possible, four males and four females from each litter. A small number of animals in each group failed to nurse their pups and were euthanised. Over the course of lactation, the dams continued to be fed the experimental diets *ad libitum*. Pups and dams were weighed daily until weaning. After post-natal day 16, food pellets were provided inside the cages to familiarise pups to the diet. On post-natal day, nineteen pups were removed from the dam and weaned onto the same experimental diets fed to the dam. At 26 d of age, the offspring were offered a mix containing equal quantities of the experimental diet and standard rat chow diet for 3 d before being weaned to standard chow diet (CRM, Special Diets Services).

Weaned pups were randomly allocated to group housing so that cages contained animals from all three maternal diet groups and fed standard rat chow diet (CRM, Special Diets Services) *ad libitum* for the remainder of the experiment. All offspring were weighed three times weekly. The body composition of the animals was measured by MRI. Blood samples were obtained from the tail vein on weeks 23, 28 and 29.

### Food intake and activity

The activity and food intake of the offspring were assessed on two separate occasions. The first measurements were conducted when the offspring were between 4 and 6 weeks of age followed by a second set of observations between 11 and 14 weeks of age.

For the first series of observations, animals were individually housed for a 7-d period in instrumented observation cages (Phenotyper, Noldus). Due to a limited number of cages, animals were randomly assigned in small batches. Food consumption was measured daily. The movement of the animals was recorded by a video camera and subsequently analysed by EthoVision XT Software (Noldus).

For the second series of observations, offspring were individually housed in cages fitted with an IR monitor system to assess activity. Food and water consumption was measured over the first 4 d. On the fourth day and for a further 3 d, a sucrose preference test was conducted using a two-bottle choice procedure. In addition to the regular water bottle, the animals were offered a second bottle containing a 1 % (wt/vol) sucrose solution. During the sucrose preference test, bottles were counterbalanced across the cages to control for side preference. The amount of sucrose and water consumed each day was calculated in g.

### Insulin tolerance test

Insulin tolerance tests were performed on the offspring at 29/30 weeks of age. Animals were fasted for 6 h, and a baseline blood sample (approximately 250 µl) was obtained by tail puncture and stored in a tube containing 3 µl of (15 %) EDTA. Insulin solution (0·75 mg/kg body weight) was administered by intraperitoneal injection. Over the following 90 min, a few drops of blood were taken from tail to measure blood glucose by the glucometer (AlphaTRAK, Abbott Laboratories). Animals continued to be monitored until blood glucose concentrations had recovered to normal levels. Data were plotted as change in glucose concentration over time, and the AUC was calculated using a trapezoidal function (Microsoft Excel).

### Homoeostatic model assessment

Animals were fasted overnight, and a blood sample was taken by tail puncture the following morning. Homoeostatic model assessment (HOMA) index was calculated by the following formula: HOMA = (glucose (mmol/l) × insulin (µmg/ml))/22·5. Glucose was measured by the glucometer, and plasma insulin was measured by the ELISA (10-1250-01 Mercodia) following the manufacturer’s instructions. Insulin concentrations were determined using the standard curve.

### Necroscopy

At 34–37 weeks of age, offspring were deeply anaesthetised with Euthatal (200 mg/ml sodium pentobarbital, Merial Animal Health) with a dose rate of 3 ml/kg body weight administered by intraperitoneal injection. Blood was collected by cardiac puncture prior to intracardial perfusion with 0·9 % NaCl solution to remove blood. Organs were removed, weighed and frozen in liquid N_2_. Samples were then stored at −70°C until analysis.

### Gene expression

Total RNA was extracted from samples of liver using RNeasy Mini Kit (Qiagen). Samples of 200 ng total RNA were reverse transcribed using the TaqMan Reverse Transcription Reagents Kit (Applied Biosystems) primed with random hexamers. The levels of cDNA were measured using custom TaqMan™ Array Cards using TaqMan® Gene Expression Assays described in online Supplementary Table 1. The relative target quantity (Rq) was calculated using the Thermo Fisher Connect Dashboard Relative Quantification qPCR Software using 18s, GAPDH and YWHAZ as internal standards.

### Statistics

Power analyses were conducted *a priori* using G * Power 3.1.9.4^(41)^. Birth weight was chosen as a primary outcome, and an effect size of 0·7 was calculated from the descriptive statistics of a previous study^(42)^. With an *α* level of 0·05, the total sample size required to achieve power of 0·8 was *n* 24 (3 groups of *n* 8). Calculations *post hoc* using the hypothesised effect size and the total sample size of 24 indicated that the actual power achieved in this study was 0·955.

Data are presented as means ± sem and analysed by ANOVA where group sizes were balanced. For logistical reasons animals were bred in three separate groups (experiments) and in some cases animals had to be further divided into separate batches for assessments, these factors together with the variability associated with animal breeding resulted in imbalanced group sizes. Data from imbalanced groups were analysed by linear mixed model (REML – Genstat 17th Edition); terms for experiment, litter size, sex, diet and diet–sex interaction formed the fixed model and dam formed the random model. If required, additional terms (weight at the start and batch) were added to the fixed model. These results are presented as predicted means ± sed.

## Results

At the start of the experiment, there were no differences in the weight or body composition of the animals (*P* > 0·05, data not shown). Animals were fed the experimental diets for a 3 week adaptation period before mating and in this time the animals fed the REC diet gained the most weight (Table 4). At the end of the adaptation period, animals fed the REC diet had approximately 20 % more body fat compared with the animals fed the AIN diet. The weight gain and body fat content of animals fed the DEP diet was intermediate between those fed the AIN and REC diets. The animals fed the DEP diet consumed approximately 8 % less food than those fed the AIN and REC diets (Table 5) and their energy intake was not different from that of the AIN fed animals despite the higher energy content of the diet. In contrast, there was no decrease in food intake in the REC fed animals and this accounted for the differences in body fat at mating.

**Table 4.** Dam characteristics during gestation (Means values with their standard error of the means)

	AIN		DEP		REC		*P*
	Mean	SEM	Mean	SEM	Mean	SEM	Diet
	*n* 22						
Pre-mating weight gain (g)	30·0^a^	3·1	35·9^a,b^	2·0	39·5^b^	2·5	0·035
Body weight at mating (g)	263·4	5·0	267·9	3·9	275·6	6·1	n.s.
Fat at mating (% body weight)	20·0^a^	0·7	21·8^a,b^	0·7	23·4^b^	0·8	0·009
Weight gain gestation days 1–7	32·2^a^	2·3	39·0^b^	2·6	35·2^a,b^	2·1	0·030
Weight gain gestation days 8–14	32·3	0·9	32·7	1·6	30·7	0·9	n.s.
Weight gain gestation days 15–21	68·3	3·2	66·8	3·4	65·7	3·0	n.s.
	*n* 7		*n* 9		*n* 8		
Litter size	12·1	1·2	12·1	1·1	14·0	1·0	n.s.
Birth weight (g)	5·0	0·2	5·2	0·1	5·3	0·2	n.s.
Sex ratio (M:F)	1·2	0·2	0·9	0·1	1·1	0·2	n.s.

All values are mean ± SEM (pre-mating *n* 22 for all groups and gestation AIN *n* 19, DEP *n* 18 and REC *n* 18.

Data are compared by one-way ANOVA.

Values with unlike superscript within rows are significantly different (*P* < 0·05). n.s. = *P* > 0·05.

**Table 5.** Dam food intake in gestation and lactation (Means values with their standard error of the means)

ANOVA	AIN		DEP		REC		*P*
	Mean	SEM	Mean	SEM	Mean	SEM	
Food intake (g/d)							
Pre-mating	18·9^b^	0·5	17·4^a^	0·3	19·1^b^	0·5	0·019
Average gestation 1–7	21·6	0·4	20·8	0·5	22·3	0·5	0·095
Average gestation 8–14	20·6	0·6	19·4	0·7	20·0	0·7	0·465
Average gestation 15–21	18·8	0·9	17·2	0·9	17·7	0·7	0·379
Average lactation 1–7	23·3	1·0	23·8	0·7	25·1	1·4	0·468
Average lactation 8–14	36·8	1·6	34·0	1·6	35·9	1·5	0·437
Average lactation 15–18	47·9^b^	2·5	41·4^a^	1·6	44·3^a,b^	1·2	0·032
Energy (kJ/d)							
Pre-mating	318·0	8·2	328·9	6·1	344·0	8·7	0·064
Average gestation 1–7	363·7^a^	7·0	392·2^b^	10·1	402·4^b^	8·5	0·006
Average gestation 8–14	346·5	10·5	366·8	13·3	361·5	11·8	0·452
Average gestation 15–21	316·7	15·3	324·4	17·3	320·1	12·3	0·935
Average lactation 1–7	392·3	16·8	450·0	12·5	453·6	24·4	0·058
Average lactation 8–14	618·7	26·3	641·3	29·3	648·4	27·8	0·760
Average lactation 15–18	806·0	42·8	781·0	31·0	799·8	21·1	0·807

All values are mean ± SEM (pre-mating *n* 22 for all groups; gestation AIN *n* 19, DEP *n* 18 and REC *n* 18 and for lactation AIN *n* 7, DEP *n* 9 and REC *n* 8).

Data are compared by one-way ANOVA.

Values with unlike superscript within rows are significantly different (*P* < 0·05).

n.s. = *P* > 0·05.

Following mating, animals in all three diet groups increased their food intake (Table 5) and in gestation week 1 consumed similar quantities of diet. As a result, the energy intake of the DEP and REC fed animals was higher than the group fed the AIN diet. Animals fed the DEP diet gained approximately 21 % more weight in the first week of gestation compared with those fed the AIN and REC diets (Table 4). However, as gestation progressed food intake fell and the total weight gain over the course of gestation was similar in all three diet groups.

**Fig. 1.**
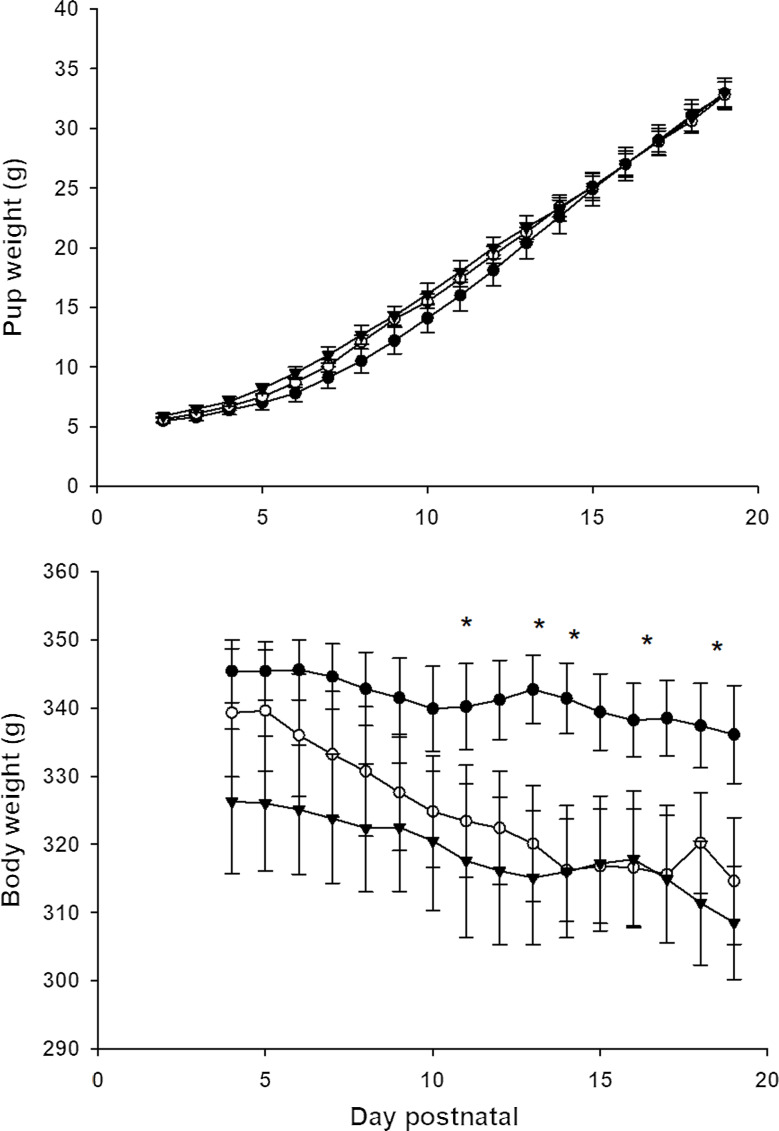
Growth in lactation. Mean body weight of pups (upper panel) and dam weight (lower panel) during the lactation phase. Closed circle AIN, closed triangle DEP and open circle REC. Error bars = SEM, AIN *n* 7, DEP *n* 9 and REC *n* 8 litters.

The birth weight of the pups, litter size and sex ratios were similar in all three groups (Table 4). There were some subtle changes in the pattern of post-natal growth of the pups (Fig. 1(a)), with the pups of dams fed AIN gaining less weight between days 7 and 8 than those of the dams fed DEP and REC diets (*P* = 0·022); however, this was temporary and the pups in the AIN group then recovered and gained more weight between days 14 and 15 (*P* = 0·023).

The food intake of the dams increased after they had given birth, with animals in all three groups having similar intakes in the first 2 weeks of lactation. In the third week of lactation, the AIN fed animals ate slightly more (Table 5). There was a tendency (*P* = 0·058) for energy intake to be higher in the DEP and REC fed animals during the first week after birth, but, thereafter, energy intakes were similar in all three groups. The body weight of the dams fed the DEP and REC diets decreased more than those fed the AIN diet (Fig. 1(b)) (repeated-measures ANOVA diet × time interaction *P* = 0·003).

There were no differences in the weight of the pups when they were weaned on post-natal day 19. A small number of male pups were killed on post-natal day 19 and dissected to measure organ weights (Table 6). The maternal diet did not change the weights of the liver, brain and kidneys; however, the hearts of pups from dams fed the REC diet were approximately 13 % heavier than those of pups from dams fed the DEP diet (*P* = 0·039). The epididymal fat pads of the pups from REC fed dams were also heavier than those of the pups from AIN fed animals with intermediate values for the DEP fed animals (*P* = 0·021).

**Table 6.** Body and organ weights of male pups at weaning

	AIN		DEP		REC		*P*
	Mean	sem	Mean	sem	Mean	sem	
Male pup weight at weaning (g)	31·6	1·74	33·0	0·91	33·7	1·15	n.s.
Liver (% body weight)	3·71	0·07	3·57	0·08	3·55	0·1	n.s.
Kidney (% body weight)	1·19	0·03	1·12	0·04	1·04	0·12	n.s.
Heart (% body weight)	0·83^a,b^	0·05	0·75^a^	0·02	0·86^b^	0·02	0·039
Brain (% body weight)	3·73	0·12	3·74	0·06	3·63	0·11	n.s.
Epididymal Fat (% body weight)	0·16^a^	0·02	0·19^a,b^	0·01	0·24^b^	0·01	0·021

All values are mean ± sem (AIN *n* 6, DEP *n* 15, REC *n* 10).

Data are compared by one-way ANOVA.

Values with unlike superscript within rows are significantly different (*P* < 0·05).

n.s. = *P* > 0·05.

### Post-natal growth and body composition of the offspring

After post-natal week 4, offspring from all three maternal diet groups were given *ad libitum* access to standard rat chow diet. At 4 weeks of age, there were no differences in the live weights of the offspring (Table 7); however, by 30 weeks of age, the weight of offspring in the DEP group was 6–8 % less than the offspring of dams fed the AIN diet. Similarly, the offspring of dams fed the REC diet weighed 9–12 % less than the animals in the AIN group. This decrease in live weight was matched by a corresponding decrease in the lean tissue mass (Table 7). At 4 weeks of age, the offspring of the REC group had the highest proportion of body fat, approximately 11 % higher than in the DEP group and approximately 19 % higher than in the AIN group (*P* < 0·001). However, this difference was transient and by 30 weeks of age animals in all three groups had similar body composition with no difference in fat as a proportion of body weight between the different maternal diet groups. Post-mortem measurements of organ weights at 37 weeks (online Supplementary Table 2) showed that the overall body weight was reflected in the absolute weights of the major organs. There were no differences in tissue weights expressed as proportions of body weight, indicating symmetrical growth of the organs in all three diet groups.

**Table 7.** Post-natal growth of the offspring

REML	Week no	Males			Females			SED	Batch	Litter size	Sex	Diet	Sex × diet
		AIN	DEP	REC	AIN	DEP	REC						
Weight (g)	4	60·2	52·5	58·0	53·6	49·9	53·5	3·6	n.s.	n.s.	<0·001	n.s.	n.s.
	10	334·7	329·3	322·0	212·0	202·4	198·3	7·1	n.s.	0·029	<0·001	n.s.	n.s.
	21	554·8^a^	536·7^a,b^	508·6^b^	279·7	257·4	255·8	12·6	<0·001	n.s.	<0·001	0·018	n.s.
	30	648·4^a^	612·6^b^	594·9^b^	314·3^a^	292·3^a,b^	279·8^b^	16·2	n.s.	n.s.	<0·001	0·028	n.s.
Fat (g)	4	8·5	7·8	9·7	7·5	7·5	9·0	0·7	n.s.	n.s.	0·002	n.s.	n.s.
	10	51·9	49·2	48·4	38·4^a^	34·0^b^	34·4^b^	1·8	0·030	0·004	<0·001	0·027	n.s.
	21	105·7	98·5	98·5	57·8^a^	46·1^b^	55·2^a^	4·5	<0·001	n.s.	<0·001	0·044	n.s.
	30	134·9	127·3	132·7	64·4	55·5	59·3	8·5	n.s.	n.s.	<0·001	n.s.	n.s.
Lean (g)	4	48·1	41·8	45·2	43·1	39·5	41·9	2·8	n.s.	n.s.	<0·001	n.s.	n.s.
	10	272·9	271·6	267·3	169·7	164·6	160·7	6·4	n.s.	n.s.	<0·001	n.s.	n.s.
	21	407·7^a^	384·3^b^	411·6^a^	215·6^a^	202·2^a,b^	191·6^b^	10·6	<0·001	n.s.	<0·001	0·041	n.s.
	30	474·9^a^	430·5^b^	450·5^a^	236·9^a^	210·2^b^	225·3^a,b^	12·8	n.s.	n.s.	<0·001	0·017	n.s.
Fat (%)	4	14·1^a^	14·8^a^	16·5^b^	13·9^a^	15·1^a^	16·7^b^	0·5	0·003	<0·001	n.s.	<0·001	n.s.
	10	15·5	15·0	15·0	18·2	16·7	17·4	0·7	0·017	n.s.	<0·001	n.s.	n.s.
	21	19·1	18·3	19·2	20·6	17·8	21·5	1·1	n.s.	n.s.	0·049	n.s.	n.s.
	30	20·7	20·9	22·3	20·6	19·0	20·6	1·7	n.s.	n.s.	n.s.	n.s.	n.s.
Fat:lean	4	0·176^a^	0·187^a^	0·212^b^	0·174^a^	0·193^a^	0·214^b^	0·007	0·002	<0·001	n.s.	<0·001	n.s.
	10	0·190	0·182	0·182	0·228	0·206	0·215	0·010	0·030	n.s.	<0·001	n.s.	n.s.
	21	0·261	0·241	0·255	0·271	0·228	0·287	0·018	n.s.	n.s.	n.s.	n.s.	n.s.
	30	0·285	0·288	0·311	0·276	0·249	0·276	0·029	n.s.	n.s.	n.s.	n.s.	n.s.

Data analysed by REML. Data are estimated means plus sed.

Numbers of animals are given in Table 3.

Values with unlike superscript within rows differ by more than 2× sed.

n.s. = *P* > 0·05.

### Food intake and activity of the offspring

The *ad libitum* food intakes of the offspring were measured on two occasions, at 4–6 weeks of age and again at 11–14 weeks of age (Table 8). In the first period, between 4 and 6 weeks of offspring of the dams fed the REC diet ate less and gained less weight than those in the AIN and DEP groups. The differences were numerically more pronounced in the female offspring of REC fed dams, which consumed approximately 15 % less food and gained approximately 20 % less weight, compared with the males where the difference was approximately 5 %. When food intake was reassessed at 11–14 weeks of age (Table 8), both weight gain and food intake were similar in the offspring from all three maternal diet groups.

**Table 8.** Offspring food intake

REML	Males			Females			sed	Batch	Wt at start	Sex	Diet	Sex × diet
	AIN	DEP	REC	AIN	DEP	REC						
4–6 weeks of age												
Wt gain (g)	52·1	51·2	48·8	38·0^a^	36·3^b^	30·4^c^	2·3	0·277	<0·001	<0·001	0·006	n.s.
Average food intake (g/d)	18·5	18·2	17·6	17·8^a^	17·0^a^	15·3^b^	0·7	<0·001	<0·001	<0·001	0·029	n.s
Mean distance – night	20 165	20 341	19 678	23 541	23 412	22 553	2199	<0·001	–	0·021	n.s.	n.s.
Mean distance – day	18 640	17 939	17 899	19 484	21 222	19 550	1883	<0·001	–	0·068	n.s.	n.s.
11–14 weeks of age												
Wt gain (g)	21·9	20·0	18·2	10·3	8·5	11·8	4·5	<0·001	<0·001	n.s.	n.s.	n.s.
Average food intake (g/d)	26·3	25·6	26·7	25·3	25·5	25·9	1·2	<0·001	<0·001	n.s.	n.s.	n.s.
Average water intake (g/d)	26·6	25·1	26·6	24·0	22·2	23·5	2·2	<0·001	<0·001	n.s.	n.s.	n.s.
Mean beam breaks/night	7029	6944	7067	8631	7960	7651	503·4	<0·001	–	<0·001	n.s.	n.s.
Mean beam breaks/d	5183	5419	5038	6223	5821	6123	364·1	<0·001	–	<0·001	n.s.	n.s.
Sucrose preference test												
Average food intake (g/d)	24·6	23·8	25·1	28·9	28·4	26·7	2·6	<0·001	<0·001	n.s.	n.s.	n.s.
Average sucrose intake g/d	35·3	32·6	37·9	41·3	38·0	41·8	6·8	0·042	<0·001	n.s.	n.s.	n.s.
Average water intake g/d	2·6	2·6	2·6	2·9	3·3	4·3	0·9	0·014	0·481	n.s.	n.s.	n.s.

Data analysed by REML.

Data are estimated means plus sed.

Numbers of animals are given in Table 3.

Values with unlike superscript within rows differ by more than 2× sed.

n.s. = *P* > 0·05.

The activity of the animals was measured at the same time as food intake by following movement via a camera placed over the cage (4–6 weeks) or by measuring breaks in an IR beam monitor (11–14 weeks). In both cases, there was no difference in the activity of animals from the different maternal diet groups or changes to the circadian pattern of activity.

When the adult animals were offered a choice of sucrose or water, the animals consumed more sucrose solution. However, there were no differences between the maternal diet groups in the absolute amount consumed or in the ratio of sucrose to water (Table 8).

### Glucose homoeostasis in the offspring

Steady-state *β* cell function and insulin sensitivity in the offspring were assessed using the HOMA calculated from fasted blood samples taken at weeks 23, 28 and 29^(43)^. Although there was some variation between the different batches of animals (Table 9) and between the male and female offspring, there were no differences due to the maternal diet. In addition, the animals were also subjected to an intraperitoneal insulin tolerance test at 29 weeks of age and the AUC is shown in Table 9. The results of the insulin tolerance test were comparable to the HOMA values and were similar for the three maternal diet groups.

**Table 9.** Glucose metabolism in the offspring

	Males			Females			sed	Batch	Sex	Diet	Sex × diet
	AIN	DEP	REC	AIN	DEP	REC					
HOMA 23 weeks	10·16	9·65	6·33	3·35	2·74	2·93	1·84	0·662	<0·001	0·237	0·164
HOMA 28 weeks	10·51	12·44	6·86	3·92	4·64	3·59	2·11	0·003	<0·001	0·122	0·146
HOMA 29 weeks	11·06	12·01	7·21	4·73	5·05	3·07	2·42	0·010	<0·001	0·150	0·598
ITT AUC (mmol/l x min)	361·8	396·5	342·0	269·4	309·1	271·8	34·0	0·042	<0·001	0·362	0·724

Data analysed by REML. Data are estimated means plus sed.

Numbers of animals are given in Table 3.

Values with unlike superscript within rows differ by more than 2× sed.

n.s. = *P* > 0·05.

### Hepatic gene expression in the offspring

To evaluate *de novo* lipogenesis and *β*-oxidation, the expression of genes involved in fat metabolism was measured in the liver of the offspring at 37 weeks of age (Table 10). The abundance of the mRNA for acetyl CoA carboxylase (Acaca) was approximately 20 % higher in the livers of offspring from dams fed the DEP diet compared with those from dams fed the REC diet, whereas the abundance of fatty acid synthase (Fasn) was unchanged. The abundance of mRNA coding for liver-type carnitine palmitoyl CoA oxidase (Cpt1a) and acyl CoA oxidase (Acox1), involved in fatty acid oxidation, was unchanged. In addition to a nearly 20-fold difference in the expression of CD36 between males and females, there was also a 35–90 % increase in expression in the offspring of both sexes from REC fed dams compared with the offspring of dams fed the DEP diet. The expression of regulators in the PPAR family was unchanged by the maternal diet; however, expression of the Srebp-1c mRNA was approximately 20 % less in offspring of dams fed the REC diet compared with the offspring of DEP fed dams.

**Table 10.** Hepatic gene expression in the offspring (Means values with their standard error of the means

	Males						Females								
	AIN		DEP		REC		AIN		DEP		REC		Two-way ANOVA		
	Mean	sem	Mean	sem	Mean	sem	Mean	sem	Mean	sem	Mean	sem	Sex	Diet	Sex × diet
Acaca	0·82	0·08	1·10	0·17	0·82	0·08	0·73	0·06	1·06	0·19	0·87	0·09	n.s.	0·048	n.s.
Fasn	0·70	0·03	1·12	0·22	0·68	0·10	1·40	0·09	1·45	0·17	1·45	0·21	<0·001	n.s.	n.s.
Cd36	1·43^a^	0·19	1·12^a^	0·10	1·52^a^	0·31	27·52^b^	2·77	20·59^b^	4·18	38·15^c^	1·95	<0·001	<0·001	0·002
Cpt1a	0·97	0·07	1·01	0·13	0·96	0·05	0·64	0·07	0·87	0·09	0·90	0·14	0·047	n.s.	n.s.
Acox1	1·08	0·06	1·28	0·17	1·03	0·08	0·75	0·03	0·92	0·11	0·98	0·07	n.s.	n.s.	n.s.
Ppara	1·10	0·10	0·91	0·05	1·01	0·05	0·48	0·05	0·71	0·15	0·58	0·05	<0·001	n.s.	n.s.
Pparg	1·57	0·13	1·27	0·27	1·34	0·13	0·59	0·06	0·67	0·06	0·54	0·06	<0·002	n.s.	n.s.
Ppargc1a	0·98	0·12	1·09	0·21	1·15	0·27	1·53	0·22	2·32	0·19	1·69	0·25	<0·003	n.s.	n.s.
Srebf1	0·98^c^	0·07	1·01^c^	0·10	0·81^b,c^	0·05	0·50^a^	0·05	0·59^a,b^	0·08	0·45^a^	0·03	<0·006	0·037	n.s.

Expression calculated as relative quantity (Rq) compared with 18s, GAPDH and YWHAZ internal standards and given in arbitrary units.

Data analysed by two-way ANOVA.

Data are estimated means ± sem (*n* 6 for each group).

Values with unlike superscript within rows are significantly different (*P* < 0·05).

n.s. = *P* > 0·05.

## Discussion

Rodents have been widely used to investigate the mechanisms linking obesity and maternal overnutrition to a subsequent increase in the risk of the offspring developing non-communicable diseases. However, it is challenging to create a reproducible experimental diet that effectively models the complex balance of macro- and micro-nutrients in human foods. Diets composed of natural products such as the cafeteria diet are unsuited to metabolic studies because animals self-select diet components with the result that each animal has a unique diet that differs from every other^(44)^. Our aim was to create a diet prepared from purified components, eliminating this variability and also taking into account the higher metabolic rate of rodents by adjusting the micronutrient content to maintain the nutrient density^(33)^. This study shows that despite a persistent reduction in the lean tissue growth of the offspring of dams fed both the DEP and REC diets, these changes did not translate into impaired glycaemic control or to increases in adiposity in the adult offspring. There was however some evidence for changes in hepatic lipid metabolism, suggesting important differences in the long-term effects on lipid metabolism.

Inevitably, the formulation of a semi-synthetic diet requires some compromises. For example, in humans a large proportion of the animal protein is from meat, so ideally part of the protein source would have a digestibility and amino acid profile corresponding to that of meat. However, to the best of our knowledge there is no suitable purified meat protein available and dried meat products are not suitable as they are of variable composition and contain endogenous lipids and minerals. As a result, we chose to use casein, a milk protein, as the animal protein component of the diet. A similar approach was also taken for the plant-based protein component of the diet, with gluten chosen in preference to soya protein as the latter may contain phyto-oestrogens. The total fibre content of the DEP diet (3·3 % by weight) corresponds to the proportion of fibre recorded in the diet diaries; however, the patterns in the human diet are more diverse and will include soluble fibre types. Soluble and insoluble fibre are both reported to be beneficial to rats, and further studies are required to understand the importance of soluble fermentable fibres in high energy diets.

Analysis of the diets as prepared showed that the fatty acid profile was as expected, including the presence of trans-fatty acids, which are relatively abundant in poor quality diets^(45)^. Unlike the AIN diet, which did not contain detectable levels of trans-fatty acids, the DEP and REC diets, respectively, contained 0·12 g and 0·05 g/100 g of diet. In the case of the DEP diet, this correspond to 0·7 % of energy from trans-fatty acids and is comparable to the levels reported for humans.

The main feature of both the DEP and REC diets is their greater energy density compared with the commonly used AIN diet. Feeding pregnant rats high-sucrose^(46)^ or cafeteria diets^(47,48)^ produces small but rather variable increases in the adiposity in the pups at weaning, suggesting that additional energy in the maternal diet is being transferred to the offspring. However, despite the DEP diet having a higher energy content, the proportion of fat as a percentage of body weight at weaning was comparable to that of pups from dams fed the AIN diet. In contrast, the weanlings of animals fed the REC diet, which also had a higher energy content than the AIN diet, was increased by approximately 20 %. Previous studies in rats^(49)^ suggest that this additional body fat in the weanlings is derived from lipid accumulated during the early stages of gestation. Although both DEP and REC diets provide more energy than the AIN diet, the DEP fed animals reduced their food intake during the pre-mating period so that the accumulation of lipid was comparable to that of animals fed the AIN diet. Although dietary fat is an important regulator of food intake in rodents^(50)^, the results suggest that additional interactions, possibly involving the differing fatty acid profiles, micronutrient composition or salt content, have differentially affected the regulation of food intake in this critical period.

The excess adiposity in the REC offspring was, however, short lived and had disappeared by 10 weeks of age. This was due to a transient decrease in food intake in the weeks immediately after weaning and once the excess fat in the REC animals had been lost, the offspring in all three groups went on to have similar proportions of fat to lean over the remainder of the experiment. The rat has a strong appetite control that regulates body composition^(51)^ and a similar short-term decrease in food intake to normalise body composition has been reported in other models of maternal overfeeding^(52)^. Systematic reviews of rodent studies have concluded that feeding obesogenic diets over the course of gestation and lactation had no effect on the appetite of the offspring^(53)^, and these results suggest that this is also true of the offpring of dams fed DEP or REC diets. Long-term effects on appetite appear to be restricted to dietary treatments, which create severe undernourishment of the dams and a much greater decrease in the growth of the offspring, for example, animals limited to 30 % of the *ad libitum* intake^(54)^, or fed low-protein diets^(55)^. Translating these results to humans would imply that changes in appetite regulation may be a consequence of stunting but not of the imbalances typical of the Western diet.

There is also evidience to suggest that the maternal diet may programme hedonistic feeding behaviours in the offspring^(56)^. For example, the offspring of dams fed cafeteria diet during pregnancy and lactation show a preference for the same range of fat, sugar and salt-rich foods in adult life^(57)^. In this study, hedonistic responses were assessed using a sucrose preference test in which animals were presented with a choice of drinking water or sucrose solution. Offspring from all three diet groups showed a marked preference for sucrose solution demonstrating a capacity to experience hedonic pleasure^(58)^. However, there were no differences between the diet groups, suggesting that the diets used in this study had no effect on the hedonistic response to sweet foods. This implies that there may be some form of conditioned behaviour induced by the cafeteria diet, for example, responding to the extremes of sweetness or the constant variety of the diet. It would be interesting to test this by conducting a more direct comparison of the cafeteria and semi-synthetic diets.

Although the ratio of fat to lean tissue in the adult offspring was unchanged by the maternal diet, there were changes in the overall growth trajectory, comparable to the effects seen in the offspring of dams fed cafeteria^(59)^ or high-fat diets^(60)^. The adult body weight of the offspring of dams fed the DEP and REC diets was approximately 10 % less than that of those fed the AIN diet. One of the main objectives in the formulation of the AIN-93G diet was to maintain the dam’s body weight in lactation^(61)^ and as a result the AIN diet contains more protein of a higher quality (20 % w/w of casein) than the DEP and REC diets (16·4 % w/w of a mixture of casein and gluten). Changes in the amino acid supply resulting from the increased protein supply in the AIN diet (Dasgin manuscript in preparation) or increased accumulation of intramuscular lipid^(59)^ may be factors affecting lean tissue growth in the period up to weaning. There may also be changes in growth trajectory of the offspring in the post-weaning period as the REC offspring reduced their food intake to compensate for the excess of adiposity at weaning. However, reducing food intake to limit energy intake has the additional effect of reducing protein intake, with a consequential effect on lean tissue growth.

A relationship between poor early growth and adult glucose metabolism is well established in animal models. For example, feeding rats a low-protein diet (the AIN 93M maintenance diet containing 8 % casein) during gestation and lactation reduces the adult body weight of the offspring by 5–15 % and changes insulin secretion and fatty acid oxidation^(62)^. However, despite a decrease in adult weight, comparable to that of the low-protein offspring, the insulin tolerance and the HOMA index of the DEP or REC offspring did not differ either from one another or from the AIN group. Changes in insulin action have also been reported in the offspring of rats fed high-fat diets; however, a systematic review^(13)^ noted a number of inconsistencies between studies. For example, some studies have used diets with much higher proportions of fat, up to 60 % of energy content from fat^(63)^ compared with 32 % in the current experiment and these unrealistic levels of dietary lipid may have exceeded the capacity of maternal metabolism to protect the offspring. A limitation of the present study is the relatively short adaptation period when the dams were fed the diet prior to mating. Elevated plasma TAG caused by diets high in sucrose^(64)^ and fat^(65)^ has been associated with the development of insulin resistance, and it is unclear whether resistance would have developed in the relatively short period when animals were fed the experimental diets before mating. It is possible that feeding the diet for a longer period prior to mating may have increased the adipose reserves of the dams and induced insulin resistance, which may, in turn, have limited the capacity to protect the developing fetus. Strain-specific effects may also be important, since the Hooded Lister strain of rats used in the present study is relatively insensitive to obesity and metabolic dysfunction.

Although physiological measurements showed no lasting changes in metabolism, there were changes in hepatic gene expression in the REC offspring consistent with altered lipid metabolism. There was a marked increase in the expression of the fatty acid translocase CD36 in the REC compared with the DEP offspring, which would be expected to facilitate the uptake of long-chain fatty acids^(66)^. At the same time, the abundance of the mRNA for SREBP-1c and one of its targets, acyl-CoA carboxylase (Acaca or Acc-1), were lower in the REC compared with the DEP offspring. As Acc-1 is the first step in the *de novo* synthesis of fatty acids^(67,68)^, these changes may be indicative of reduced lipogenesis. Overall, these results are consistent with an increased hepatic utilisation of fatty acids with a concomitant reduction in *de novo* lipogenesis by the REC offspring. It has been suggested that a shift in metabolism, which promotes lipid utilisation at the expense of glucose, may reduce the development of insulin resistance^(69)^.

Although there were no interactions between diet and the sex of the offspring in relation to growth or glucose metabolism, sex-specific effects were aparent in the expression of CD36. A similar change in the expression of genes of lipid metabolism specific to the female offspring of mice fed a high fat Western diet was observed in microarray studies of hepatic gene expression^(70)^. Since the expression of CD36 is much higher in females due to its stimulation by oestrogen and growth hormone^(71)^, there may be underlying sex-specific effects of maternal diet on lipid metabolism mediated through the female sex hormones.

In conclusion, although numerous studies of high-fat diets suggest long-term changes in glycaemic control and adiposity in the offspring^(13)^, the present results suggest that diets that replicate many of the features of socio-economically deprived human diets produce more limited effects. Despite changes in growth and adiposity, maternal metabolic adaptation minimises the adverse consequences of the imbalanced maternal diet on the metabolism of the offspring, limiting them to changes in hepatic gene expression. Since the changes in gene expression are indirect measures of lipid metabolism, it will be interesting to make more direct measurements in these offspring and to extend the studies to other tissues including skeletal and cardiac muscle. Since exposure to obesogenic diets in the real world does not cease at weaning, these changes in gene expression may also modify the response of the offspring to a high-fat or high-sucrose challenge in adult life.

## Supporting information

Dasgin et al. supplementary material Dasgin et al. supplementary material

## Supplementary material

For supplementary material referred to in this article, please visit https://doi.org/10.1017/S0007114523002210

## References

[1] Hanson MA & Gluckman PD (2014) Early developmental conditioning of later health and disease: physiology or pathophysiology? Physiol Rev 94, 1027–1076. 25287859 10.1152/physrev.00029.2013 PMC4187033

[2] Stanner SA , Bulmer K , Andres C , et al. (1997) Does malnutrition in utero determine diabetes and coronary heart disease in adulthood? Results from the Leningrad siege study, a cross sectional study. BMJ 315, 1342–1348. 9402775 10.1136/bmj.315.7119.1342 PMC2127836

[3] Roseboom T , de Rooij S & Painter R (2006) The Dutch famine and its long-term consequences for adult health. Early Hum Dev 82, 485–491. 16876341 10.1016/j.earlhumdev.2006.07.001

[4] Forsen T , Eriksson JG , Tuomilehto J , et al. (1999) Growth in utero and during childhood among women who develop coronary heart disease: longitudinal study. BMJ 319, 1403–1407. 10574856 10.1136/bmj.319.7222.1403 PMC28284

[5] Osmond C , Barker D , Winter P , et al. (1993) Early growth and death from cardiovascular disease in women. BMJ 307, 1519–1524. 8274920 10.1136/bmj.307.6918.1519 PMC1679586

[6] Barker DJ (2007) The origins of the developmental origins theory. J Intern Med 261, 412–417. 17444880 10.1111/j.1365-2796.2007.01809.x

[7] Godfrey KM & Barker DJ (2000) Fetal nutrition and adult disease. Am J Clin Nutr 71, 1344s–1352s. 10799412 10.1093/ajcn/71.5.1344s

[8] Cordain L , Eaton SB , Sebastian A , et al. (2005) Origins and evolution of the western diet: health implications for the 21st century. Am J Clin Nutr 81, 341–354. 15699220 10.1093/ajcn.81.2.341

[9] Haggarty P , Campbell DM , Duthie S , et al. (2009) Diet and deprivation in pregnancy. Br J Nutr 102, 1487–1497. 19682400 10.1017/S0007114509990444

[10] Food Standards Agency Scotland. The Scottish Diet – It Needs to Change 2018 Update. https://www.foodstandards.gov.scot/publications-and-research/publications/the-scottish-diet-it-needs-to-change-2018-update2018 (accessed October 2023).

[11] Parisi F , Laoreti A & Cetin I (2014) Multiple micronutrient needs in pregnancy in industrialized countries. Ann Nutr Metab 65, 13–21. 25227491 10.1159/000365794

[12] UNICEF (2019) Children, Food and Nutrition: Growing Well in a Changing World. The State of the World’s Children. https://www.unicef.org/reports/state-of-worlds-children-2019 (accessed October 2023).

[13] Ainge H , Thompson C , Ozanne SE , et al. (2011) A systematic review on animal models of maternal high fat feeding and offspring glycaemic control. Int J Obes 35, 325–335. 10.1038/ijo.2010.149 20680016

[14] Ribaroff GA , Wastnedge E , Drake AJ , et al. (2017) Animal models of maternal high fat diet exposure and effects on metabolism in offspring: a meta-regression analysis. Obes Rev 18, 673–686. 28371083 10.1111/obr.12524 PMC5434919

[15] Williams L , Seki Y , Vuguin PM , et al. (2014) Animal models of in utero exposure to a high fat diet: a review. Biochim Biophys Acta 1842, 507–519. 23872578 10.1016/j.bbadis.2013.07.006 PMC3895417

[16] Fernandez-Twinn DS , Wayman A , Ekizoglou S , et al. (2005) Maternal protein restriction leads to hyperinsulinemia and reduced insulin signalling protein expression in 21 month-old female rat offspring. Am J Physiol Regul Integr Comp Physiol 288, R368–R373. 15514105 10.1152/ajpregu.00206.2004

[17] Berends LM , Dearden L , Tung YCL , et al. (2018) Programming of central and peripheral insulin resistance by low birthweight and postnatal catch-up growth in male mice. Diabetologia 61, 2225–2234. 30043179 10.1007/s00125-018-4694-z PMC6133152

[18] Wargent ET , Martin-Gronert MS , Cripps RL , et al. (2020) Developmental programming of appetite and growth in male rats increases hypothalamic serotonin (5-HT)5A receptor expression and sensitivity. Int J Obes 44, 1946–1957. 10.1038/s41366-020-0643-2 32719434

[19] Butruille L , Marousez L , Pourpe C , et al. (2019) Maternal high-fat diet during suckling programs visceral adiposity and epigenetic regulation of adipose tissue stearoyl-CoA desaturase-1 in offspring. Int J Obes 43, 2381–2393. 10.1038/s41366-018-0310-z 30622312

[20] Ozanne SE , Dorling MW , Wang CL , et al. (2000) Depot-specific effects of early growth retardation on adipocyte insulin action. Horm Metab Res 32, 71–75. 10741689 10.1055/s-2007-978592

[21] Cerf ME & Herrera E (2016) High fat diet administration during specific periods of pregnancy alters maternal fatty acid profiles in the near-term rat. Nutrients 8, 25. 26742067 10.3390/nu8010025 PMC4728639

[22] Ashino NG , Saito KN , Souza FD , et al. (2012) Maternal high-fat feeding through pregnancy and lactation predisposes mouse offspring to molecular insulin resistance and fatty liver. J Nutr Biochem 23, 341–348. 21543214 10.1016/j.jnutbio.2010.12.011

[23] Gray C , Harrison CJ , Segovia SA , et al. (2015) Maternal salt and fat intake causes hypertension and sustained endothelial dysfunction in fetal, weanling and adult male resistance vessels. Sci Rep 5, 9753. 25953742 10.1038/srep09753 PMC4424661

[24] Segovia SA , Vickers MH , Harrison CJ , et al. (2018) Maternal high-fat and high-salt diets have differential programming effects on metabolism in adult male rat offspring. Front Nutr 5, 1. 29564328 10.3389/fnut.2018.00001 PMC5845870

[25] Kirk SL , Samuelsson AM , Argenton M , et al. (2009) Maternal obesity induced by diet in rats permanently influences central processes regulating food intake in offspring. PLOS ONE 4, e5870. 19516909 10.1371/journal.pone.0005870 PMC2690656

[26] Pereira TJ , Fonseca MA , Campbell KE , et al. (2009) Maternal obesity characterized by gestational diabetes increases the susceptibility of rat offspring to hepatic steatosis via a disrupted liver metabolome. J Physiol 593, 3181–3197. 10.1113/JP270429 PMC4532536 25922055

[27] Samuelsson AM , Matthews PA , Argenton M , et al. (2008) Diet-induced obesity in female mice leads to offspring hyperphagia, adiposity, hypertension, and insulin resistance: a novel murine model of developmental programming. Hypertension 51, 383–392. 18086952 10.1161/HYPERTENSIONAHA.107.101477

[28] Blackmore HL , Niu Y , Fernandez-Twinn DS , et al. (2014) Maternal diet-induced obesity programs cardiovascular dysfunction in adult male mouse offspring independent of current body weight. Endocrinology 155, 3970–3980. 25051449 10.1210/en.2014-1383 PMC4255219

[29] Speakman JR (2019) Use of high-fat diets to study rodent obesity as a model of human obesity. Int J Obes 43, 1491–1492. 10.1038/s41366-019-0363-7 30967607

[30] Rees WD (2019) Interactions between nutrients in the maternal diet and the implications for the long-term health of the offspring. Proc Nutr Soc 78, 88–96. 30378511 10.1017/S0029665118002537

[31] Davis RE & Icke GC (1983) Clinical chemistry of thiamin. Adv Clin Chem 23, 93–140. 6398618 10.1016/s0065-2423(08)60399-6

[32] Ramakrishnan U , Goldenberg T & Allen LH (2011) Do multiple micronutrient interventions improve child health, growth, and development? J Nutr 141, 2066–2075. 21956959 10.3945/jn.111.146845

[33] Newmark HL (1987) Nutrient density: an important and useful tool for laboratory animal studies. Carcinogenesis 8, 871–873. 3594720 10.1093/carcin/8.7.871

[34] Hintze KJ , Benninghoff AD & Ward RE (2012) Formulation of the total western diet (TWD) as a basal diet for rodent cancer studies. J Agric Food Chem 60, 6736–6742. 22224871 10.1021/jf204509a

[35] Beverley B , David, C , Kerry S , et al. (2020) National Diet and Nutrition Survey Rolling Programme Years 9 to 11 (2016/2017 to 2018/2019) – A Survey Carried Out on Behalf of Public Health England and the Food Standards Agency. PHE. https://www.repository.cam.ac.uk/handle/1810/334369 (accessed October 2023).

[36] Vennemann FBC , Ioannidou S , Valsta LM , et al. (2015) Dietary intake and food sources of choline in European populations. Br J Nutr 114, 2046–2055. 26423357 10.1017/S0007114515003700

[37] Scientific Advisory Committee on Nutrition (2012) Dietary Reference Values for Energy. HM Stationery Office.

[38] Dietary reference values for food energy and nutrients for the United Kingdom: report of the Panel on Dietary Reference Values of the Committee on Medical Aspects of Food Policy. HM Stationery Office; 1991. 1961974

[39] Institute of Medicine (1998) Standing Committee on the Scientific Evaluation of Dietary Reference Intakes and its Panel on Folate, Other B Vitamins, and Choline. Dietary Reference Intakes for Thiamin, Riboflavin, Niacin, Vitamin B_6_, Folate, Vitamin B_12_, Pantothenic Acid, Biotin, and Choline. Washington, DC: The National Academy Press. 23193625

[40] Lobley GE , Bremner DM , Holtrop G , et al. (2007) Impact of high-protein diets with either moderate or low carbohydrate on weight loss, body composition, blood pressure and glucose tolerance in rats. Br J Nutr 97, 1099–1108. 17397561 10.1017/S0007114507691934

[41] Faul F , Erdfelder E , Lang AG , et al. (2007) G*Power 3: a flexible statistical power analysis program for the social, behavioral, and biomedical sciences. Behav Res Methods 39, 175–191. 17695343 10.3758/bf03193146

[42] Maloney CA , Hay SM & Rees WD (2009) The effects of feeding rats diets deficient in folic acid and related methyl donors on the blood pressure and glucose tolerance of the offspring. Br J Nutr 101, 1333–1340. 18782463 10.1017/S0007114508066798

[43] Antunes LC , Elkfury JL , Jornada MN , et al. (2016) Validation of HOMA-IR in a model of insulin-resistance induced by a high-fat diet in Wistar rats. Arch Endocrinol Metab 60, 138–142. 27191048 10.1590/2359-3997000000169

[44] Moore BJ (1987) The cafeteria diet – an inappropriate tool for studies of thermogenesis. J Nutr 117, 227–231. 3550006 10.1093/jn/117.2.227

[45] Hutchinson J , Rippin HL , Jewell J , et al. (2017) Comparison of high and low trans-fatty acid consumers: analyses of UK national diet and nutrition surveys before and after product reformulation. Public Health Nutr 21, 465–479. 29157320 10.1017/S1368980017002877 PMC10260737

[46] Morahan HL , Leenaars CHC , Boakes RA , et al. (2021) Metabolic and behavioural effects in offspring exposed to maternal sucrose consumption: a systematic review and meta-analysis of data from rodent models. J Dev Orig Health Dis 12, 603–618. 32907667 10.1017/S2040174420000823

[47] Vithayathil MA , Gugusheff JR , Ong ZY , et al. (2018) Exposure to maternal cafeteria diets during the suckling period has greater effects on fat deposition and sterol regulatory element binding protein-1c (SREBP-1c) gene expression in rodent offspring compared to exposure before birth. Nutr Metab 15, 17. 10.1186/s12986-018-0253-3 PMC5815184 29467799

[48] George G , Draycott SAV , Muir R , et al. (2019) The impact of exposure to cafeteria diet during pregnancy or lactation on offspring growth and adiposity before weaning. Sci Rep 9, 14173. 31578441 10.1038/s41598-019-50448-x PMC6775089

[49] Zambrano E , Martínez-Samayoa PM , Rodríguez-González GL , et al. (2010) RAPID REPORT: dietary intervention prior to pregnancy reverses metabolic programming in male offspring of obese rats. J Physiol 588, 1791–1799. 20351043 10.1113/jphysiol.2010.190033 PMC2887995

[50] Hu S , Wang L , Yang D , et al. (2018) Dietary fat, but not protein or carbohydrate, regulates energy intake and causes adiposity in mice. Cell Metab 28, 415–431. 30017356 10.1016/j.cmet.2018.06.010

[51] Cohn C & Joseph D (1962) Influence of body weight and body fat on appetite of “normal” lean and obese rats. Yale J Biol Med 34, 598–607. 13880343 PMC2604224

[52] Toop CR , Muhlhausler BS , O’Dea K , et al. (2017) Impact of perinatal exposure to sucrose or high fructose corn syrup (HFCS-55) on adiposity and hepatic lipid composition in rat offspring. J Physiol 595, 4379–4398. 28447343 10.1113/JP274066 PMC5491864

[53] Lagisz M , Blair H , Kenyon P , et al. (2015) Little appetite for obesity: meta-analysis of the effects of maternal obesogenic diets on offspring food intake and body mass in rodents. Int J Obes 39, 1669–1678. 10.1038/ijo.2015.160 26293233

[54] Vickers MH , Breier BH , Cutfield WS , et al. (2000) Fetal origins of hyperphagia, obesity, and hypertension and postnatal amplification by hypercaloric nutrition. Am J Physiol Endocrinol Metab 279, E83–E87. 10893326 10.1152/ajpendo.2000.279.1.E83

[55] Cripps RL , Martin-Gronert MS , Archer ZA , et al. (2009) Programming of hypothalamic neuropeptide gene expression in rats by maternal dietary protein content during pregnancy and lactation. Clin Sci 117, 85–93. 10.1042/CS20080393 19152506

[56] Wright TM , Fone KC , Langley-Evans SC , et al. (2011) Exposure to maternal consumption of cafeteria diet during the lactation period programmes feeding behaviour in the rat. Int J Dev Neurosci 29, 785–793. 22004940 10.1016/j.ijdevneu.2011.09.007

[57] Bayol SA , Farrington SJ & Stickland NC (2007) A maternal ‘junk food’ diet in pregnancy and lactation promotes an exacerbated taste for ‘junk food’ and a greater propensity for obesity in rat offspring. Br J Nutr 98, 843–851. 17697422 10.1017/S0007114507812037

[58] Berridge KC (2004) Motivation concepts in behavioral neuroscience. Physiol Behav 81, 179–209. 15159167 10.1016/j.physbeh.2004.02.004

[59] Bayol SA , Simbi BH & Stickland NC (2005) A maternal cafeteria diet during gestation and lactation promotes adiposity and impairs skeletal muscle development and metabolism in rat offspring at weaning. J Physiol 567, 951–961. 16020464 10.1113/jphysiol.2005.088989 PMC1474235

[60] Visco DB , Manhães-de-Castro R , da Silva MM , et al. (2020) Early life fluoxetine treatment causes long-term lean phenotype in skeletal muscle of rats exposed to maternal lard-based high-fat diet. Biomed Pharmacother 131, 110727. 32927255 10.1016/j.biopha.2020.110727

[61] Reeves PG , Nielsen FH & Fahey GC (1993) AIN-93 purified diets for laboratory rodents: final report of the American Institute of Nutrition writing committee on the reformulation of the AIN-76A rodent diet. J Nutr 123, 1939–1951. 8229312 10.1093/jn/123.11.1939

[62] Agnoux AM , Antignac J-P , Simard G , et al. (2014) Time window-dependent effect of perinatal maternal protein restriction on insulin sensitivity and energy substrate oxidation in adult male offspring. Am J Physiol Regul Integr Comp Physiol 307, R184–R197. 24808498 10.1152/ajpregu.00015.2014

[63] Tamashiro KLK , Terrillion CE , Hyun J , et al. (2009) Prenatal stress or high-fat diet increases susceptibility to diet-induced obesity in rat offspring. Diabetes 58, 1116–1125. 19188431 10.2337/db08-1129 PMC2671057

[64] Soria A , Chicco A , Mocchiutti N , et al. (1996) A sucrose-rich diet affects triglyceride metabolism differently in pregnant and nonpregnant rats and has negative effects on fetal growth. J Nutr 126, 2481–2486. 8857508 10.1093/jn/126.10.2481

[65] Storlien LH , Jenkins AB , Chisholm DJ , et al. (1991) Influence of dietary fat composition on development of insulin resistance in rats. Relationship to muscle triglyceride and *n*-3 fatty acids in muscle phospholipid. Diabetes 40, 280–289. 1991575 10.2337/diab.40.2.280

[66] Glatz JC & Luiken JFP (2018) Dynamic role of the transmembrane glycoprotein CD36 (SR-B2) in cellular fatty acid uptake and utilization. J Lipid Res 59, 1084–1093. 29627764 10.1194/jlr.R082933 PMC6027920

[67] Mao J , DeMayo FJ , Li H , et al. (2006) Liver-specific deletion of acetyl-CoA carboxylase 1 reduces hepatic triglyceride accumulation without affecting glucose homeostasis. Proc Natl Acad Sci USA 103, 8552–8557. 16717184 10.1073/pnas.0603115103 PMC1570106

[68] Mao J , Yang T , Gu Z , et al. (2009) aP2-Cre-mediated inactivation of acetyl-CoA carboxylase 1 causes growth retardation and reduced lipid accumulation in adipose tissues. Proc Natl Acad Sci USA 106, 17576–17581. 19805143 10.1073/pnas.0909055106 PMC2762677

[69] Cheung L , Andersen M , Gustavsson C , et al. (2007) Hormonal and nutritional regulation of alternative CD36 transcripts in rat liver – a role for growth hormone in alternative exon usage. BMC Mol Biol 8, 60. 17640331 10.1186/1471-2199-8-60 PMC1934915

[70] Mischke M , Pruis MG , Boekschoten MV , et al. (2013) Maternal western-style high fat diet induces sex-specific physiological and molecular changes in 2-week-old mouse offspring. PLOS ONE 8, e78623. 24223833 10.1371/journal.pone.0078623 PMC3818485

[71] Ståhlberg N , Rico-Bautista E , Fisher RM , et al. (2004) Female-predominant expression of fatty acid translocase/CD36 in rat and human liver. Endocrinology 145, 1972–1979. 14684613 10.1210/en.2003-0874

[72] Reeves PG (1997) Components of the AIN-93 diets as improvements in the AIN-76A diet. J Nutr 127, Suppl. 5, 838S–841S. 9164249 10.1093/jn/127.5.838S

