# Supplementary material for: Diet and deprivation in pregnancy: a rat model to investigate the effects of the maternal diet on the growth of the dam and its offspring
Source: Br J Nutr. 2023 Oct 5;131(4):630–41. doi: 10.1017/S0007114523002210 (PMC10803821; doi:10.1017/S0007114523002210)
Supplement: Dasgin et al. supplementary material [file S0007114523002210sup001.docx]

Supplementary Table 1

QPCR primers

| Gene symbol | Assay reference |
| --- | --- |
| Acaca | Rn00573474_m1 |
| Fasn | Rn00569117_m1 |
|  |  |
| Cd36 | Rn00580728_m1 |
| Cpt1a | Rn00580702_m1 |
| Acox1 | Rn01460628_m1 |
|  |  |
| Ppara | Rn00566193_m1 |
| Pparg | Rn00440945_m1 |
| Ppargc1a | Rn00580241_m1 |
| Srebf1 | Rn01495769_m1 |
|  |  |
| 18S | Hs99999901_s1 |
| Gapdh | Rn99999916_s1 |
| Ywhaz | Rn00755072_m1 |

Supplementary Table 2

Offspring organ weights at post mortem

| REML |  | Males |  |  | Females |  |  |  |  |  |  |
| --- | --- | --- | --- | --- | --- | --- | --- | --- | --- | --- | --- |
|  | AIN | SIMD | SACN | AIN | SIMD | SACN | sed | Expt | Gender | Diet | Gender.Diet |
| Body wt | 653.8 | 613.0 | 602.8 | 308.2 | 278.2 | 276.5 | 20.6 | 0.025 | <0.001 | 0.034 | 0.782 |
| Liver | 22.840 | 21.600 | 20.070 | 11.360 | 10.620 | 9.480 | 0.994 | 0.941 | <0.001 | 0.026 | 0.784 |
| Heart | 1.794 | 1.677 | 1.717 | 1.186 | 1.094 | 1.085 | 0.060 | 0.007 | <0.001 | 0.045 | 0.855 |
| L Kidney | 2.079 | 2.198 | 2.067 | 1.356 | 1.285 | 1.192 | 0.102 | 0.013 | <0.001 | 0.297 | 0.360 |
| R Kidney | 2.227 | 2.220 | 2.092 | 1.391 | 1.324 | 1.212 | 0.102 | 0.059 | <0.001 | 0.117 | 0.909 |
| Brain | 1.898 | 1.906 | 1.860 | 1.662 | 1.682 | 1.634 | 0.034 | 0.021 | <0.001 | 0.291 | 0.946 |
| Soleus | 0.256 | 0.228 | 0.218 | 0.139 | 0.131 | 0.113 | 0.011 | <0.001 | <0.001 | 0.002 | 0.444 |
| Plantaris | 0.476 | 0.460 | 0.416 | 0.267 | 0.277 | 0.245 | 0.023 | 0.091 | <0.001 | 0.034 | 0.479 |
| Gastrocnemius | 2.226 | 2.143 | 2.068 | 1.249 | 1.188 | 1.131 | 0.072 | 0.003 | <0.001 | 0.083 | 0.903 |
| Fat | 9.354 | 8.967 | 9.743 | 4.096 | 3.365 | 3.849 | 0.849 | 0.772 | <0.001 | 0.595 | 0.834 |
| Total fat | 18.350 | 17.320 | 18.590 | 7.890 | 5.540 | 7.480 | 1.310 | 0.012 | <0.001 | 0.221 | 0.752 |
| Testis/Ovary | 2.900 | 2.498 | 2.921 | 0.213 | 0.214 | 0.183 | 0.164 | 0.240 | <0.001 | 0.198 | 0.075 |

Data analysed by REML Data are estimated means plus sed. Numbers of animals are given in Table 3. Values with unlike superscript within rows differ by more than 2x sed. n.s. = p>0.05
